# Crystal structure of Prp16 in complex with ADP

**DOI:** 10.1107/S2053230X23005721

**Published:** 2023-07-25

**Authors:** Tim Benedict Garbers, Marieke Enders, Piotr Neumann, Ralf Ficner

**Affiliations:** a Department of Molecular Structural Biology, Institute of Microbiology and Genetics, GZMB, Georg-August-University Göttingen, Göttingen, Germany; Sungkyunkwan University School of Medicine, Republic of Korea

**Keywords:** DEAH-box helicases, Prp16, *Chaetomium thermophilum*, spliceosome

## Abstract

DEAH-box helicases play a crucial role in pre-mRNA splicing as they are responsible for major rearrangements of the spliceosome and are involved in various quality-ensuring steps. Prp16 is the driving force during spliceosomal catalysis, remodeling the C state into the C* state. Here, the first crystal structure of Prp16 from *Chaetomium thermophilum* in complex with ADP is reported at 1.9 Å resolution. Comparison with the other spliceosomal DEAH-box helicases Prp2, Prp22 and Prp43 reveals an overall identical domain architecture. The β-hairpin, which is a structural element of the RecA2 domain, exhibits a unique position, punctuating its flexibility. Analysis of cryo-EM models of spliceosomal complexes containing Prp16 reveals that these models show Prp16 in its nucleotide-free state, rendering the model presented here the first structure of Prp16 in complex with a nucleotide.

## Abstract 2

Prp16 is a DEAH-box ATPase required for the splicing of pre-mRNA. The X-ray crystal structure of the Prp16-ADP complex was determined at a resolution of 1.9 Å.

## 1. Introduction

The spliceosome is large molecular machinery that is responsible for the removal of noncoding introns from eukaryotic precursor messenger RNAs (pre-mRNAs). For each intron removal, the spliceosome is assembled *de novo* and performs two subsequent transesterification reactions. In the course of each cycle, the components assemble in a stepwise manner and a number of conformational changes take place (Will & Lührmann, 2011 ▸). The spliceosome consists of five small nuclear ribonucleoprotein complexes (snRNPs) and several other non-snRNP proteins (Wahl *et al.*, 2009 ▸). One cycle can be divided into assembly, activation, catalysis and disassembly stages. The first event in each cycle is the binding of the U1 snRNP to the 5′-splice site of the pre-mRNA, forming the E complex. Binding of the U2 snRNP to the branch-point sequence and recruitment of the pre-assembled tri-snRNP U4/U6/U5 conclude the assembly step, forming the pre-B complex. For the activation of the spliceosome, the U1 and the U4 snRNPs are displaced while the NineTeen complex (NTC) and the NTC-related complex (NTR) bind, forming the catalytically competent B* spliceosome. Following the first transesterification reaction, the resulting C complex is further remodeled into the C* complex, which can facilitate the second transesterification reaction. Finally, the spliceosome is disassembled, releasing the remaining snRNPs, NTC and NTR, the severed intron lariat and the spliced mRNA (Ficner *et al.*, 2017 ▸). The structural rearrangements of the spliceosome include RNA–RNA, protein–RNA and protein–protein remodeling, which need to be tightly orchestrated and to undergo rigorous quality-control steps, as misspliced mRNA can be linked to diseases such as Duchenne muscular dystrophy (Takeshima *et al.*, 2010 ▸) and spinal muscular atrophy (Lorson *et al.*, 1999 ▸). All of these remodeling steps are driven and controlled by the action of RNA helicases belonging to helicase superfamily 2 (SF2; Cordin & Beggs, 2013 ▸). The SF2 helicases involved in splicing can be further divided into three subfamilies: DEAD-box, DEAH-box and Ski2-like helicases (Jankowsky & Fairman, 2007 ▸).

The protein studied in this work is pre-mRNA-splicing factor 16 (Prp16) and is required for the transition of the spliceosome from the C to the C* state (Wilkinson *et al.*, 2021 ▸). Therefore, Prp16 binds the 3′-end sequence of the intron RNA and translocates towards the spliceosome, leading to dissociation of the branching factors Yju2 and Cwc25 and thereby enabling exon ligation (Tseng *et al.*, 2011 ▸). The translocation also plays a role in splicing-fidelity control in a kind of kinetic proofreading mechanism where Prp16 antagonizes suboptimal substrates and promotes optimal substrates for 5′-splice site cleavage (Burgess & Guthrie, 1993 ▸; Koodathingal *et al.*, 2010 ▸; Koodathingal & Staley, 2013 ▸; Semlow *et al.*, 2016 ▸). Prp16 belongs to the DEAH-box proteins, which share a highly conserved helicase core formed by two RecA-like domains. These two domains provide the necessary architecture that enables DEAH-box helicases to be functional NTPases (Schwer & Guthrie, 1992 ▸). The nucleotide binds at the domain interface, where the eight conserved sequence motifs I, Ia, Ib, II, III, IV, V and VI are located. Motifs I, II and VI are involved in nucleic acid binding of the helicase and motif II contains the eponymous sequence DEAH. While motifs Ia, Ib and IV are necessary to bind and hydrolyze the nucleoside triphosphate, motif III is required to couple NTP hydrolysis to the process of unwinding (Campodonico & Schwer, 2002 ▸; Schneider *et al.*, 2004 ▸). All DEAH-box helicases have a common C-terminal domain architecture comprising winged-helix (WH), helix-bundle (HB) and oligosaccharide-binding fold (OB-fold) domains. These domains have a regulatory effect on the hydrolyzation rate of the helicase (Kudlinzki *et al.*, 2012 ▸). It has also been shown that this part can serve as a platform for the binding of interaction partners (Cordin & Beggs, 2013 ▸). The N-terminal regions of the spliceosomal DEAH-box helicases greatly vary in their length and are the least conserved region (Cordin & Beggs, 2013 ▸).

Recent cryo-EM structures (three-dimensional reconstructions) of spliceosomal complexes in the C or C* state from human or yeast contain map areas which can be related to parts of or even complete Prp16 molecules (Wilkinson *et al.*, 2021 ▸; Galej *et al.*, 2016 ▸; Yan *et al.*, 2017 ▸; Bertram *et al.*, 2020 ▸; Zhan *et al.*, 2018 ▸). All atomic models of the Prp16 structures were modeled based on the structurally related yeast Prp43 in complex with ADP (PDB entries 3kx2 and 2xau; He *et al.*, 2010 ▸; Walbott *et al.*, 2010 ▸). As the local resolution of the cryo-EM maps at the position of Prp16 ranges between 8 and 15 Å, the derived atomic models of Prp16 provide only limited information about the structure of this helicase.

Here, we report the first crystal structure of Prp16 from the thermophilic eukaryotic ascomycete *Cheatomium thermophilum* in its ADP-bound state at 1.9 Å resolution. Analysis of the structure shows the same domain architecture as observed for other spliceosomal DEAH-box helicases. Moreover, the interaction pattern of Prp16 with ADP also seems to be conserved within this helicase family. In addition, a new position of the β-hairpin could be observed, punctuating its proposed flexibility.

## 2. Materials and methods

### 2.1. Macromolecule production

The Prp16-encoding gene of *C. thermophilum* (*ct*Prp16) was identified by the NCBI *BLAST* search tool (Sequence ID EGS23320.1). The codon-optimized sequence encoding *ct*Prp16 residues 302–920 was cloned into the pGEX-6P-3 vector utilizing the BamHI and EcoRI restriction sites. The recombinant protein was expressed using *Escherichia coli* Rosetta2 (DE3) cells in 2×YT medium. The cells were induced with 0.5 m*M* isopropyl β-d-1-thioglactopyranoside at an optical density (OD_600_) of 0.6 and were incubated at 16°C for 20 h. The cells were harvested and washed with phosphate-buffered saline. Prior to cell disruption via a microfluidizer, the cells were mixed at 4 ml g^−1^ with a buffer consisting of 50 m*M* Tris–HCl pH 7.5, 500 m*M* NaCl, 10 m*M* EDTA. Subsequently, the lysate was centrifuged at 20 000*g* and 4°C for 30 min. The supernatant was applied onto a Glutathione Sepharose 4B column (GE Healthcare). A washing step with additional 2 *M* LiCl was performed to remove bound nucleotides and nucleic acids. Finally, the protein was eluted using 30 m*M* reduced glutathione. Cleavage of the GST tag was performed by incubation with PreScission protease [100:1(*w*:*w*)]. Further impurities and the GST tag were removed by applying the protein onto a Superdex 200 gel-filtration column (GE Healthcare) coupled to a Glutathione Sepharose 4B column (GE Healthcare) in 10 m*M* Tris–HCl pH 7.5, 200 m*M* NaCl, 2 m*M* MgCl_2._ A final check for remaining nucleotides was performed by measuring the *A*_260_/*A*_280_ absorbance. The protein was concentrated to 20 mg ml^−1^ using an Amicon Ultra centrifugal concentrator (Merck). A total of 8 mg *ct*Prp16 (302–920) could be obtained from 2 l medium. The protein solution was aliquoted into PCR tubes in 105 µl samples, flash-frozen in liquid nitrogen and stored at −80°C for further usage. Macromolecule-production information is summarized in Table 1 ▸.

### 2.2. Crystallization

Crystallization was performed by the sitting-drop vapor-diffusion method at 293 K. *ct*Prp16 (302–920) was diluted to 4 mg ml^−1^ (55.98 µ*M*) with gel-filtration buffer. To obtain *ct*Ptp16–ADP complex crystals, the protein was mixed with a tenfold molar excess of ADP (559.8 µ*M*) and reservoir solution consisting of 20%(*v*/*v*) PEG 4000 , 100 m*M* MES pH 6.5, 5 m*M* MgCl_2_. The total volume of the drop was 2 µl with a 1:1 ratio of protein and reservoir solution. Crystals were observed after one week. Crystallization information is summarized in Table 2 ▸.

### 2.3. Data collection and processing

For cryoprotection, the *ct*Prp16–ADP complex crystals were transferred to mother liquor containing an additional 10%(*w*/*v*) PEG 4000 and 550.8 µ*M* ADP before being plunged into liquid nitrogen. Oscillation diffraction images were collected on beamline P13 at DESY, Hamburg, Germany. Data were processed using *XDS* (Kabsch, 2010 ▸). Data-collection and processing statistics are summarized in Table 3 ▸.

### 2.4. Structure solution and refinement

The structure of *ct*Prp16 (302–920) was solved by molecular replacement using *Phaser* (McCoy *et al.*, 2007 ▸). The structure of *ct*Prp2 in complex with ADP (PDB entry 6fac; Schmitt *et al.*, 2018 ▸) was used as the search model. Initial rounds of refinement were performed using *Phenix* (Liebschner *et al.*, 2019 ▸). Manual model building was performed using *Coot* (Emsley *et al.*, 2010 ▸) and the model was subsequently refined using *REFMAC* (Murshudov *et al.*, 2011 ▸). No *I*/σ(*I*) cutoff was applied during refinement. Final validation of the model was conducted via *MolProbity* (Chen *et al.*, 2010 ▸). All figures were prepared using *PyMOL* (version 1.8; Schrödinger). Refinement statistics are summarized in Table 4 ▸.

## 3. Results and discussion

**Figure 1:**
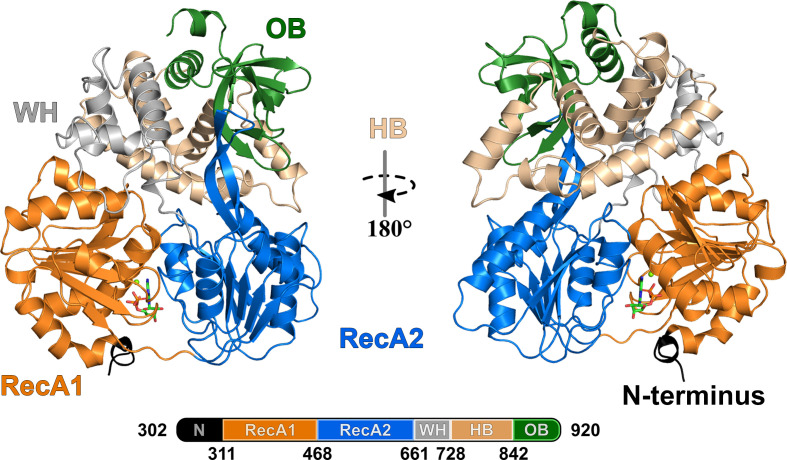
Crystal structure of *ct*Prp16 (302–920) in complex with ADP at 1.9 Å resolution. The N-terminal extension (302–311) is depicted in black, the RecA1-like domain (312–468) in orange, the RecA2-like domain (469–661) in marine blue, the WH domain (662–728) in gray, the HB domain (729–842) in wheat and the OB-fold domain (843–920) in green. The structure is depicted in cartoon mode, while the bound ADP is represented as sticks and the magnesium ion is shown as a sphere.

The spliceosomal DEAH-box helicase Prp16 from the thermo­philic ascomycete *C. thermophilum* (*ct*Prp16) was crystallized in complex with ADP to mimic a post-catalytic state. The ortholog from *C. thermophilum* was chosen as proteins from this organism exhibit high thermostability and therefore tend to crystallize better (Amlacher *et al.*, 2011 ▸). Several studies of the closely related DEAH-box helicases Prp43, Prp2 and Prp22 have shown that orthologs from *C. thermophilum* are highly suitable for structural investigations of spliceosomal helicases (Tauchert *et al.*, 2016 ▸, 2017 ▸; Schmitt *et al.*, 2018 ▸; Hamann *et al.*, 2019 ▸; Absmeier *et al.*, 2020 ▸). An N- and C-terminally truncated construct *ct*Prp16 (302–920) was used for crystallization, as the removed parts are expected to be mainly disordered according to the *PredictProtein* server (Bernhofer *et al.*, 2021 ▸). The crystal belonged to the ortho­rhombic space group *P*2_1_2_1_2_1_, with unit-cell parameters *a* = 55.13, *b* = 102.14, *c* = 106.61 Å, α = β = γ = 90° (Table 2 ▸). The phase problem was solved via molecular replacement using the structure of ADP-bound *ct*Prp2 as the search model (PDB entry 6fac). The asymmetric unit contains one *ct*Prp16 molecule. The polder omit map clearly reveals the presence of ADP and a magnesium ion in the active center (Supplementary Figs. S2 and S3). The reported structure of *ct*Prp16 in complex with ADP was determined at a resolution of 1.9 Å and was refined to an *R*_work_ of 19.3% and an *R*_free_ of 23.8%. According to the Ramachandran plot, 96.93% of all residues are in the most favored region, 3.07% are in the allowed region and 0% are outliers (Table 4 ▸). The atomic model comprises *ct*Prp16 (302–920), one ADP molecule, one magnesium ion, one polyethylene glycol (PEG) molecule and 423 water molecules. The overall assembly of *ct*Prp16 can be divided into six different entities: the N-terminal extension (302–311), two RecA-like domains [referred to as RecA1 (312–468) and RecA2 (467–661)], the WH domain (662–728), the HB domain (729–842) and the OB-fold domain (843–920) (Fig. 1 ▸). While the RecA domain architecture is shared by all members of the SF2 helicase family, the three C-terminal domains are characteristic of DEAH-box helicases. A prominent β-hairpin, which protrudes out of the RecA2 domain and comprises residues 601–620 in *ct*Prp16, is another typical feature of DEAH-box helicases.

### 3.1. ADP binding of *ct*Prp16

**Figure 2:**
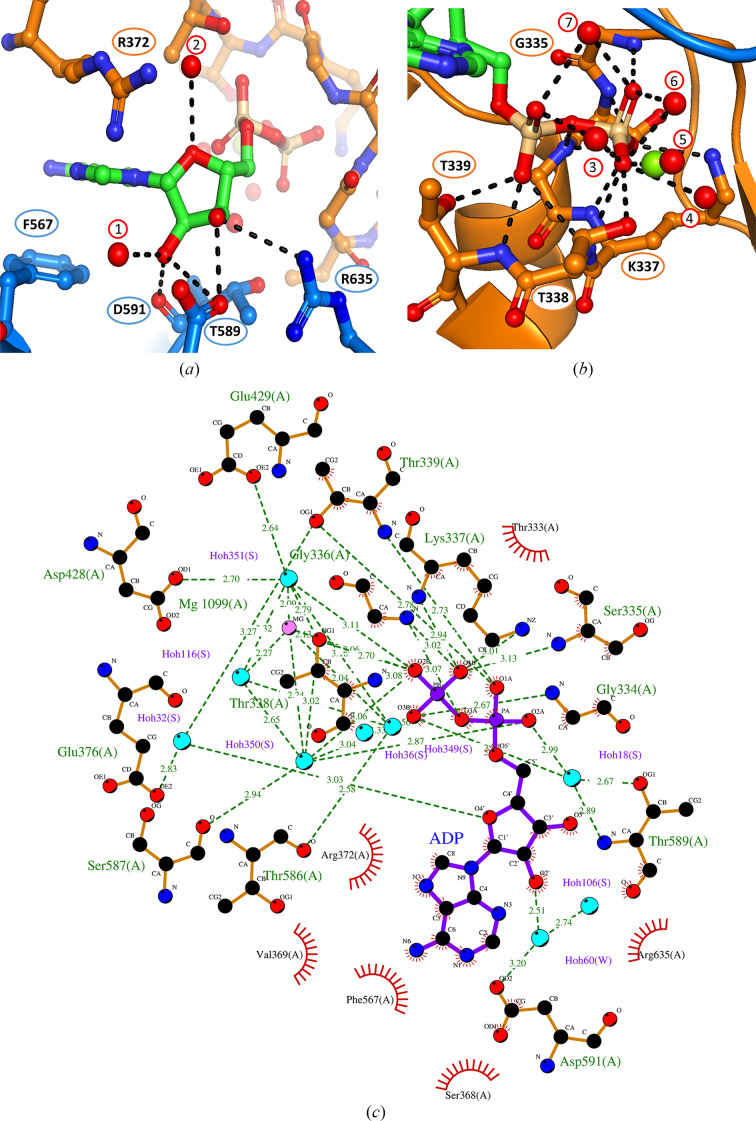
ADP-binding site of *ct*Prp16. The ADP molecule is sandwiched between the RecA1 (orange) and RecA2 (marine) domains (color scheme as in Fig. 1 ▸). C atoms are shown in orange/marine (protein) and green (ADP), N atoms are in blue, O atoms are in red, P atoms are in wheat, the Mg^2+^ ion is in light green and water molecules are shown as red spheres. Residues which are involved in ADP, water or Mg^2+^ binding are presented in ball-and-stick mode and are labeled according to the *ct*Prp16 sequence. Polar interactions are visualized as dashed black lines. (*a*) The adenine moiety is bound via π–π stacking and the ribose by hydrogen bonding. (*b*) The α- and β-phosphates participate intensively in hydrogen bonding. The central Mg^2+^ ion is coordinated by four water molecules, the Thr342 side chain and an O atom of the β-phosphate. (*c*) A two-dimensional ligand–protein interaction diagram (created with *LigPlot*+) depicting the network of interactions between the ADP molecule and the RecA1 and RecA2 domains of Prp16.

*ct*Prp16, which is a member of the SF2 helicase family, possesses a well conserved catalytic core comprising several highly similar structural motifs that are spread over both RecA-like domains. Motifs Ia (^359^TQPRRVAA^366^), Ib (^405^TDGVLLR^411^) and IV (^520^LVFMTG^525^) are reported to be necessary for interaction with the substrate nucleic acid, while motifs I (^334^GSGKT^338^), II (^428^DEAH^431^), V (^581^TNIAETSLT^589^) and VI (^628^QRAGRAGR^635^) are needed for binding and hydrolysis of the nucleoside triphosphate. Motif III (^460^SAT^462^) couples nucleotide hydrolysis to the unwinding activity. In the structure reported here, an ADP molecule as well as the catalytically required magnesium ion are present at the interface between the RecA1 and RecA2 domains, corresponding to *ct*Prp16 in the post-catalytic state. The magnesium ion is hexavalently coordinated by four water molecules, Thr342 located within motif I and the β-phosphate of the ADP molecule. The β-phosphate is furthermore coordiated by interactions with the backbone amides of Gly334, Ser335, Gly336, Lys337 and Thr338 and with the side chains of Lys341 and Thr342. The former group of five amino acids together form motif I. Additionally, four water molecules could be identified in the structure which are involved in the interaction network of the β-phosphate, making a total of 13 interactions between this phosphate and *ct*Prp16 (Fig. 2 ▸ *a*). The O2′ atom of the ribose interacts with the carbonyl backbone of Thr589, one water molecule and the side chain of Asp595. The O3′ atom interacts with the side chains of Asp595 and Arg635. An additional interaction could be observed between one water molecule and the O4′ atom of the ribose ring. While Arg635 (motif VI) belongs to this conserved region of DEAH-box helicases, Thr593 and Asp595 do not. The adenine moiety does not form any polar contacts with *ct*Prp16 residues, but the adenine interacts with Phe567 via π–π stacking (Fig. 2 ▸ *b*). This interaction seems to be highly conserved as it can be found in all available X-ray structures of spliceosomal DEAH-box helicases bound to ADP, except for one structure (PDB entry 6faa), in which the adenine base is flipped over in a *syn* conformation (Tauchert *et al.*, 2016 ▸; Schmitt *et al.*, 2018 ▸; Hamann *et al.*, 2020 ▸). The eponymous motif II of this protein class, DEAH, is not directly involved in any interactions between the protein and the nucleotide. Instead, it is involved in the formation of the interaction network of the magnesium ion by interacting with two water molecules via the carbonyl groups of Asp428 and Glu429. Furthermore, Glu131 stabilizes the position of one water molecule near the magnesium ion. Overall, the interactions of the *C. thermophilum* spliceosomal DEAH-box helicases with ADP seem to be highly conserved as in other ADP-bound helicases: *ct*Prp2 (PDB entry 6fac) and *ct*Prp43 (PDB entry 5d0u) exhibit the same interaction pattern (Schmitt *et al.*, 2018 ▸; Tauchert *et al.*, 2016 ▸).

### 3.2. Position of the β-hairpin

Based on structural comparison with the Ski2-like helicase Hel308, the RecA2 β-hairpin has been suggested to be involved in the double-strand separation of DEAH-box helicases (Büttner *et al.*, 2007 ▸; He *et al.*, 2010 ▸; Walbott *et al.*, 2010 ▸). In contrast, a crystal structure of Prp22 in complex with ssRNA shows the 5′ end of the RNA locked in a conformation with the bases pointing away from the β-hairpin, positioning a potential double strand on the opposite side of this structural element. Comparison with other helicases suggests that the conformation of the β-hairpin observed in Prp16 would support its role as a physical barrier, separating the 3′ portion of the bound RNA that exhibits a stacked conformation from the 5′ portion that mainly interacts with the C-terminal domains. Thereby, it is thought to prevent backsliding of the RNA strand during translocation (Hamann *et al.*, 2019 ▸).

**Figure 3:**
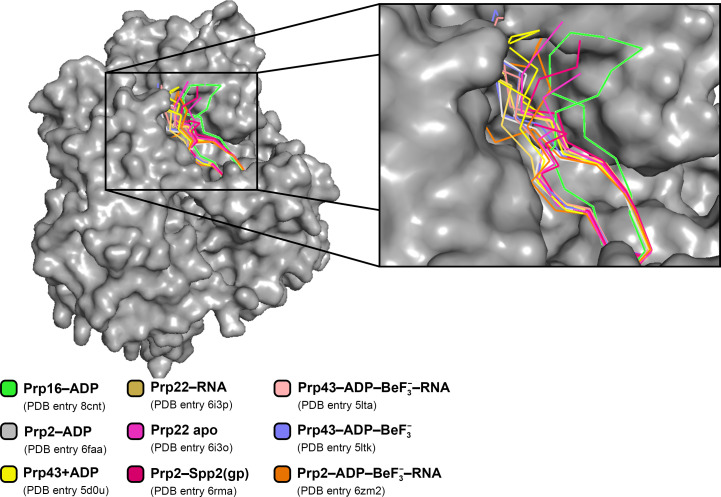
Comparison of the different β-hairpin conformations. The structure of *ct*Prp16 was superposed with different spliceosomal DEAH-box helicases from *C. thermophilum* in different catalytic states via their RecA1 domains. The different β-hairpins are represented in cartoon mode, while the remaining part of the protein is shown as a gray surface (*ct*Prp16–ADP only). Parts of the OB-fold domain were omitted for clarity. The superposition reveals that the β-hairpin of ctPrp16–ADP adapts a more distant conformation compared with the other structures.

Superpositioning of all spliceosomal DEAH-box helicase structures from *C. thermophilum* in different conformational stages reveals the unique conformation of the β-hairpin in the *ct*Prp16 structure (Fig. 3 ▸). While all other X-ray structures harbor the β-hairpin between the WH and OB-fold domains, independent of the nucleotide- or substrate-loading state, in *ct*Prp16 the upper part of the β-hairpin with its loop region is pushed out of the cleft and interacts exclusively with the OB-fold domain. However, this conformation appears to be an artifact caused by interaction with a symmetry mate within the crystal lattice (Supplementary Fig. S1). The bulky amino acids Glu306 and Phe311 of the symmetry mate, located at the N-terminus of the truncated *ct*Prp16, interfere with the loop which connects the two β-strands forming the β-hairpin. Arg610 in particular, which is in the middle of the loop region, would clash with the previously mentioned N-terminal residues if the β-hairpin adopts a position between the WH and OB-fold domains. In the known complex structures of DEAH-box helicases with bound RNA, there is only one conserved Lys residue of the β-hairpin that interacts with the RNA, for example Lys835 in the *ct*Prp22–RNA complex (Hamann *et al.*, 2019 ▸). Superposition of the *ct*Prp16 structure with the structure of the *ct*Prp22–RNA complex shows that the conserved Lys is located virtually in the same position in both structures, meaning that RNA binding seems to be unaffected by the unique conformation of the β-hairpin in *ct*Prp16.

### 3.3. Comparison with Prp16 structures emerging from cryo-EM 3D reconstructions

**Figure 4:**
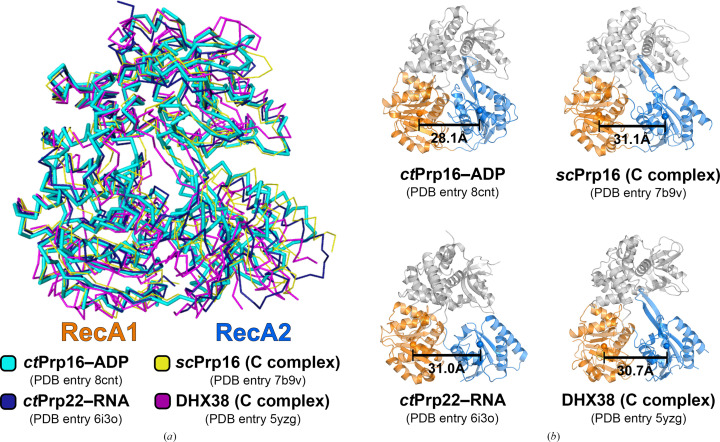
Movement of RecA2 upon nucleotide binding. (*a*) Comparison of Prp16 models originating from X-ray diffraction and cryo-EM and Prp22 (in a nucleotide-free state). The different structures are shown as ribbons and are aligned via their RecA1-like domains. The RecA2 domain of Prp16–ADP is shifted towards the RecA1 domain compared with the other structures. (*b*) All structures are depicted as semi-transparent cartoon models. The RecA1 domain is colored orange, the RecA2 domain marine and the C-terminal part gray. The centers of mass of the RecA-like domains are displayed as spheres and colored accordingly. In order to calculate the centers of mass for the same sets of atoms, the RecA1 and RecA2 domains were superimposed and the centers of mass of these superimposed domains were determined. Prp16 structures derived from a cryo-EM model adopt a more open conformation that is comparable to the distances of Prp22 in a nucleotide-free state (PDB entry 6i3o) and the mammalian ortholog DHX38 of yeast Prp16 (bottom right).

In recent years, five different structural models of spliceosomal C* or C complexes from *Saccharomyces cerevisiae* and *Homo sapiens* containing Prp16 have been obtained by means of single-particle cryo-EM (Wilkinson *et al.*, 2021 ▸; Galej *et al.*, 2016 ▸; Yan *et al.*, 2017 ▸; Bertram *et al.*, 2020 ▸; Zhan *et al.*, 2018 ▸). In the area of the spliceosome which was predicted to harbor Prp16, the authors claimed a local resolution ranging between 8 and 15 Å. Interestingly, in none of the structures was an additional cryo-EM map observed for the 300 N-terminal residues. In fact, the cryo-EM map implicated the presence of the helicase core of Prp16 or only parts of it. The N-terminal region of Prp16 could not be traced despite its function in the spliceosomal context (Wang & Guthrie, 1998 ▸). For a direct structural comparison with *ct*Prp16 in complex with ADP, the cryo-EM 3D reconstructions for which a subnanometre local resolution of Prp16 was reported were chosen (PDB entries 7b9v and 5yzg). For further comparison, the structure of the closely related spliceosomal DEAH-box helicase Prp22 in its respective apo state was also used (PDB entry 6i3o). The overall alignment shows that the RecA1 and C-terminal domains are quite similar in their arrangement, while the location of the RecA2 domain differs dramatically. In contrast to the other structures, the RecA2 domain of ADP-bound *ct*Prp16 is clearly shifted towards the RecA1 domain (Fig. 4 ▸ *a*). To compare the exact distances between the RecA-like domains of each analyzed helicase, the center of mass of each RecA domain was determined and the distance between them was measured (Fig. 4 ▸ *b*). While the structure of ADP-bound *ct*Prp16 exhibits a center-of-mass distance of 28.1 Å between the two RecA domains, the distances calculated for Prp16 molecules derived from the cryo-EM structures of spliceosomes are increased by about 3 Å (31.1 Å in PDB entry 7b8v and 30.7 Å in PDB entry 5yzg). Therefore, it can be concluded that Prp16 in the spliceosome structures is unlikely to exhibit the ADP-bound post-catalytic state. In fact, the increased RecA distance fits better to the center-of-mass distance measured in the structure of Prp22 in its apo state (PDB entry 6io3, 31.0 Å). This is in line with the suggestion of Wilkinson and coworkers, who stated that they used the structure of scPrp43 in complex with ADP as the starting model but the corresponding map was more similar to the conformation of Prp22 bound to RNA found in PDB entry 6i3p (Wilkinson *et al.*, 2021 ▸; PDB entry 7b9v). Zhan and coworkers also used the structure of scPrp43 bound to ADP as the starting model but recognized that the RecA2 domain is shifted by a distance of 5–8 Å (Zhan *et al.*, 2018 ▸; PDB entry 5yzg). Therefore, it can be concluded that the model of Prp16 presented in this work is the first model of Prp16 with subnanometre resolution bound to a nucleotide.

## Supplementary Material

PDB reference: Prp16, complex with ADP, 8cnt

Supporting information. DOI: 10.1107/S2053230X23005721/ek5032sup1.pdf

## Figures and Tables

**Table 1:** Macromolecule-production information

Source organism	*C. thermophilium* var. *thermophilum* DSM 1495
DNA source	Codon-optimized synthetic DNA
Forward primer	CGGGATCCCTGAAAGAACAGCGTGAATTTCTGCC
Reverse primer	GGAATTCTTATACGGCGGAGGGTTCCAGTTCTGCCAGCCAATGCG
Cloning vector	pETM-13
Expression vector	pGEX-6P-3
Expression host	*Escherichia coli* Rosetta 2 (DE3)
Complete amino-acid sequence of the construct produced	**GPLGSPNT**LKEQREFLPAFAVREDLLRVIRDNQVVIVIGETGSGKTTQLTQFLYEDGYGKTGMIGCTQPRRVAAMSVAKRVAEEMEVKLGTLVGYAIRFEDCTSKETVIKYMTDGVLLRESLNEPDLDRYSCIIMDEAHERALNTDVLMGLFKKILQRRRDLKLIITSATMNSKRFSDFFGGAPEFTIPGRTFPVDILFHRSPVEDYVDQAVQQVLAIHVSKPAGDILVFMTGQEDIEVTCELIQERLAALNDPPKLSVLPIYSQMPADLQAKIFDRAPPGVRKCIVATNIAETSLTVDGIMYVVDCGYSKLKVYNPRMGMDTLQITPISQANAAQRAGRAGRTGPGQAYRLYTEKQFRDEMYMQTIPEIQRTNLSNTVLLLKSLGVKDLLDFDFMDPPPQDTITTSLYDLWALGALDNLGELTELGRKMNAFPMDPPLAKLLIMSEEYGCSEEMVTIVSMLSVPNVFYRPKERQEESDAAREKFFVPESDHLTYLHVYTQWKANGYSDAWCARHFLHSKSLRRAREVRDQLLDIMKMQHMRMVSCGTDWDIIRKCICSGYYHQAAKVKGIGEYINLRTSVTVQLHPTSALYGLGFLPDYVVYHELILTSKEYMSTVTAVDPHWLAEL**EPSAV**

**Table 2:** Crystallization

Method	Vapor diffusion
Plate type	Sitting drop
Temperature (K)	293
Protein concentration	4
Buffer composition of protein solution	10 m*M* Tris–HCl pH 7.5, 200 m*M* NaCl, 2 m*M* MgCl_2_
Composition of reservoir solution	20%(*w*/*v*) PEG 4000, 100 m*M* MES pH 6.5, 5 m*M* MgCl_2_
Volume and ratio of drop	2 µl, 1:1 ratio
Volume of reservoir (µl)	500

**Table 3:** Data collection and processing

Diffraction source	P13, DESY
Wavelength (Å)	0.97625
Temperature (K)	100
Detector	EIGER 16M
Crystal-to-detector distance (mm)	217.62
Rotation range per image (°)	0.15
Total rotation range (°)	360
Exposure time per image (s)	0.04
Space group	*P*2_1_2_1_2_1_
*a*, *b*, *c* (Å)	55.13, 102.14, 106.61
α, β, γ (°)	90, 90, 90
Mosaicity (°)	0.301
Resolution range (Å)	48.97–1.90 (2.00–1.90)
Total No. of reflections	647348 (91387)
No. of unique reflections	91538 (13012)
Completeness (%)	99.9 (99.8)
Multiplicity	7.1 (7.0)
〈*I*/σ(*I*)〉†	11.8 (1.34)†
*R*_meas_	0.115 (1.686)
CC_1/2_	0.99 (0.50)
Overall *B* factor from Wilson plot (Å^2^)	31.3

† The 〈*I*/σ(*I*)〉 value in the outer shell is 1.34, but the other values are still satisfactory as CC_1/2_ = 50%.

**Table 4:** Structure refinement

Resolution range (Å)	48.97–1.90 (1.95–1.90)
Completeness (%)	99.9
No. of reflections, working set	45747 (3280)
No. of reflections, test set	2409 (173)
Final *R*_cryst_	0.193 (0.321)
Final *R*_free_	0.238 (0.385)
Cruickshank DPI	0.167
No. of non-H atoms	
Protein	4926
Ion	1
ADP	27
PEG	19
Water	432
R.m.s. deviations	
Bond lengths (Å)	0.008
Angles (°)	1.486
Average *B* factors (Å^2^)	
Protein	36
Ion	31
ADP	41
PEG	65
Ramachandran plot	
Most favored (%)	96.93
Allowed (%)	3.07
