# Supplementary material for: Crystal structure of Prp16 in complex with ADP
Source: Acta Crystallogr F Struct Biol Commun. 2023 Jul 25;79(Pt 8):200–7. doi: 10.1107/S2053230X23005721 (PMC10416764; doi:10.1107/S2053230X23005721)
Supplement: Supplementary file 1 [file f-79-00200-sup1.pdf]

STRUCTURAL BIOLOGY COMMUNICATIONS

Volume 79 (2023)

Supporting information for article:

Crystal structure of Prp16 in complex with ADP

Tim Benedict Garbers, Marieke Enders, Piotr Neumann and Ralf Ficner

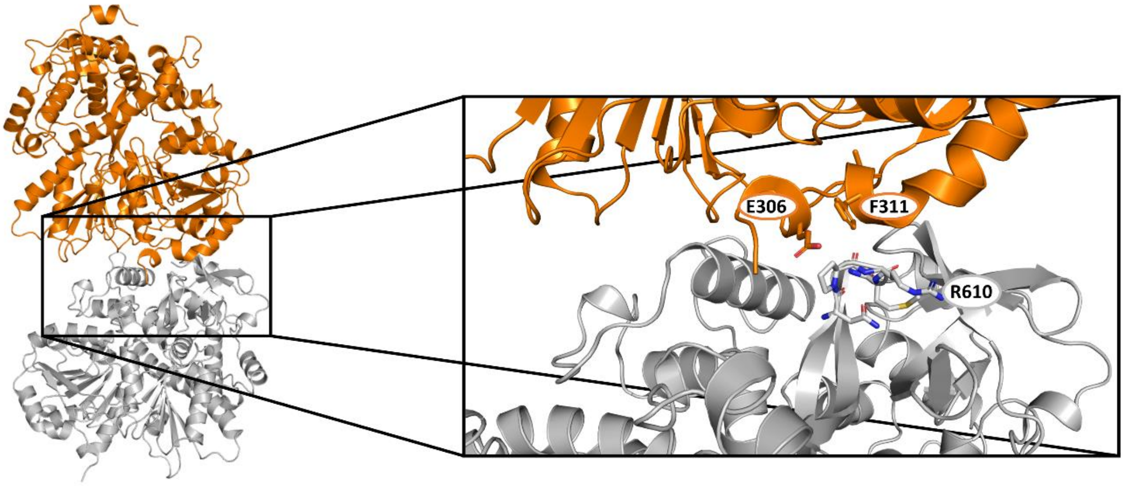

Figure S1 Interaction of Prp16 with a symmetry mate. Prp16 molecules are shown as cartoon and coloured in grey and orange. Residues of the N-terminal part and of the loop region between the β-strands forming the β-hairpin are depicted as sticks and N-atoms are coloured in blue while O-atoms are coloured in red.

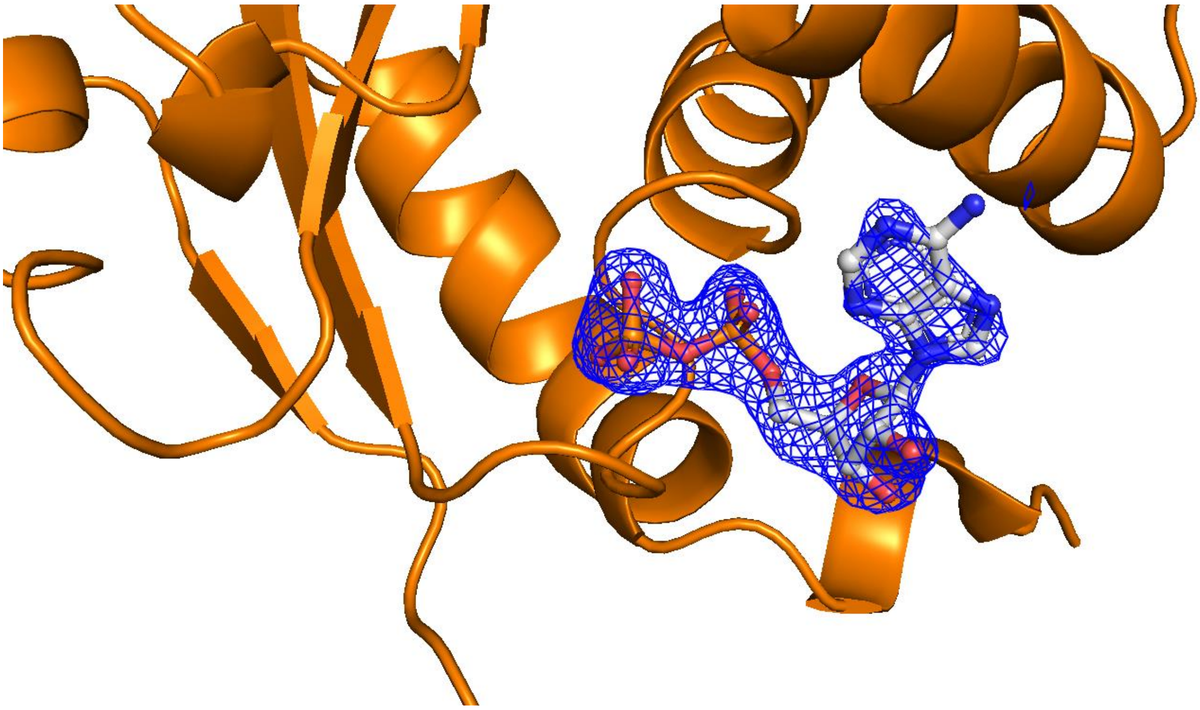

Figure S2 Polder omit map of ADP. The |Fo-Fc| electron density map is contoured at 3 σ is depicted for the ADP molecule (shown as sticks and balls) bound Prp16. The protein is depicted as cartoon and coloured in orange. Parts of the RecA2-like domain are omitted.

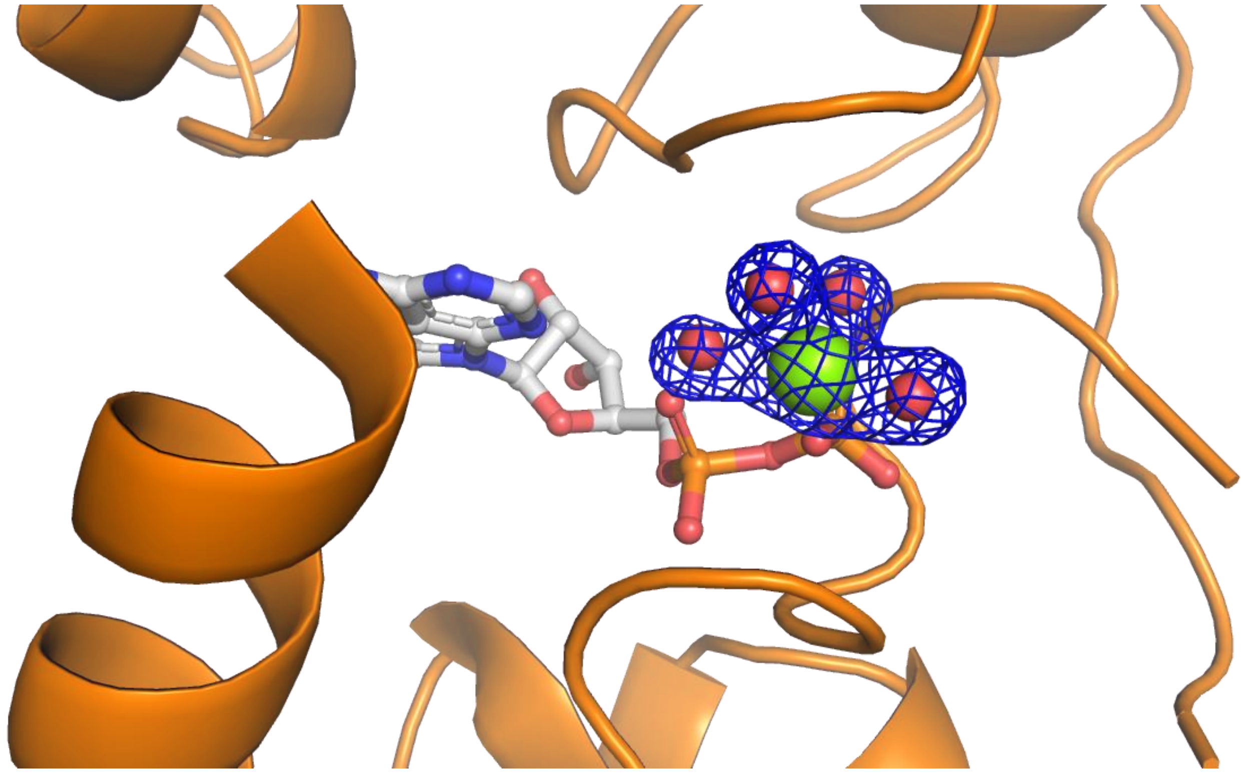

Figure S3 Polder omit map of water molecules which are part of magnesium interaction network. The |Fo-Fc| electron density map is contoured at 4 σ is depicted for the magnesium (green sphere) and four water molecules (red sphere). Prp16 is depicted as cartoon and coloured in orange. Parts of the RecA2-like domain are omitted.
